# Light Modulation of Human Clocks, Wake, and Sleep

**DOI:** 10.3390/clockssleep1010017

**Published:** 2019-03-13

**Authors:** Abhishek S. Prayag, Mirjam Münch, Daniel Aeschbach, Sarah L. Chellappa, Claude Gronfier

**Affiliations:** 1 Lyon Neuroscience Research Center (CRNL), Integrative Physiology of the Brain Arousal Systems (Waking) Team, Inserm UMRS 1028, CNRS UMR 5292, Université Claude Bernard Lyon 1, Université de Lyon, F-69000 Lyon, France; 2 Charité University Medicine Berlin, Institute of Medical Immunology, Group Chronobiology, 10117 Berlin, Germany; 3 St. Hedwig Krankenhaus, 10115 Berlin, Germany; 4 Sleep/Wake Research Centre, Massey University, Wellington 6140, New Zealand; 5 Department of Sleep and Human Factors Research, Institute of Aerospace Medicine, German Aerospace Center (DLR), 51170 Cologne, Germany; 6 Division of Sleep Medicine, Harvard Medical School, Boston, MA 02115, USA; 7 Medical Chronobiology Program, Division of Sleep and Circadian Disorders, Departments of Medicine and Neurology, Brigham and Women’s Hospital, Boston, MA 02115, USA

**Keywords:** non-image forming, light, wake, sleep, EEG activity, melanopsin, circadian, alertness and ipRGCs, melatonin

## Abstract

Light, through its non-imaging forming effects, plays a dominant role on a myriad of physiological functions, including the human sleep–wake cycle. The non-image forming effects of light heavily rely on specific properties such as intensity, duration, timing, pattern, and wavelengths. Here, we address how specific properties of light influence sleep and wakefulness in humans through acute effects, e.g., on alertness, and/or effects on the circadian timing system. Of critical relevance, we discuss how different characteristics of light exposure across the 24-h day can lead to changes in sleep–wake timing, sleep propensity, sleep architecture, and sleep and wake electroencephalogram (EEG) power spectra. Ultimately, knowledge on how light affects sleep and wakefulness can improve light settings at home and at the workplace to improve health and well-being and optimize treatments of chronobiological disorders.

## 1. Light Modulation of Non-Image Forming Functions 

### 1.1. The Eye, Vision, and Non-Image Forming Functions

For the mammalian central nervous system, light absorption is carried out by a unique interface, the retina. Photon capture by specialized photoreceptors (rods, cones, and intrinsically photosensitive retinal ganglion cells (ipRGCs)) in the retina is conveyed to the brain for visual processing and non-image forming (a.k.a. nonvisual) responses. The processing of light information for vision is conveyed via different photoreceptor classes in the outer retina (S-, M-, L-cones and rods) and delivered to the brain via the optic nerve. In addition to rods and cones, the ipRGCs, located in the inner retina, convey light information mainly for non-image forming functions (NIF), such as sleep–wake and circadian rhythm regulation. The ipRGCs integrate and transmit photic information via the retino-hypothalamic tract (RHT) directly to the suprachiasmatic nuclei (SCN) [1,2], thereby entraining the circadian timing system to the daily 24-h light/dark cycle [3]. Besides the SCN, ipRGCs also project to a diverse set of nuclei in the hypothalamus and subcortical limbic zones, including the lateral geniculate complex, the habenular regions, the superior colliculus, the olivary pretectal nucleus, the pericacqueductal gray, and the ventrolateral preoptic area (VLPO) [4,5]. As such, light conveyed by ipRGCs has direct effects on a variety of NIF functions (e.g., circadian rhythms, pupil light reflex, sleep–wake cycle), [6,7,8,9,10,11,12,13] and also on vision [14,15,16,17,18,19] in humans, and animals. Different ipRGCs subtypes might underpin the range of NIF functions influenced by light [20,21].

### 1.2. Acute and Circadian Effects of Light

The circadian system is orchestrated by a master clock, located in the SCN of the hypothalamus [22], and entrained to the 24-h light-dark cycle via light exposure. The master clock in turn synchronizes peripheral clocks located in other tissues outside the SCN, e.g., in the liver, lung, muscle, retina, kidney, or cortex [3]. The effects of light on the circadian timing system are well characterized even though there is new evidence in nocturnal animals that ipRGC projections to the SCN can mediate the effects of light, for example on learning, independently from the SCN pacemaker function [23]. Light also impacts acutely (direct) a range of non-image forming functions in animal and humans, such as alertness and cognitive processes, sleep/wake cycle, pupillary light reflex, heart rate, body temperature, melatonin secretion [8,9,13,24,25,26]. These functions, as well as the circadian system, encode several properties of light, which we discuss in the following sections. We also describe functions related to sleep and circadian physiology. An overview of the important references discussed in the following sections is provided in Table 1.

#### 1.2.1. Differences in Spectral Sensitivity to Light

**Figure 1:**
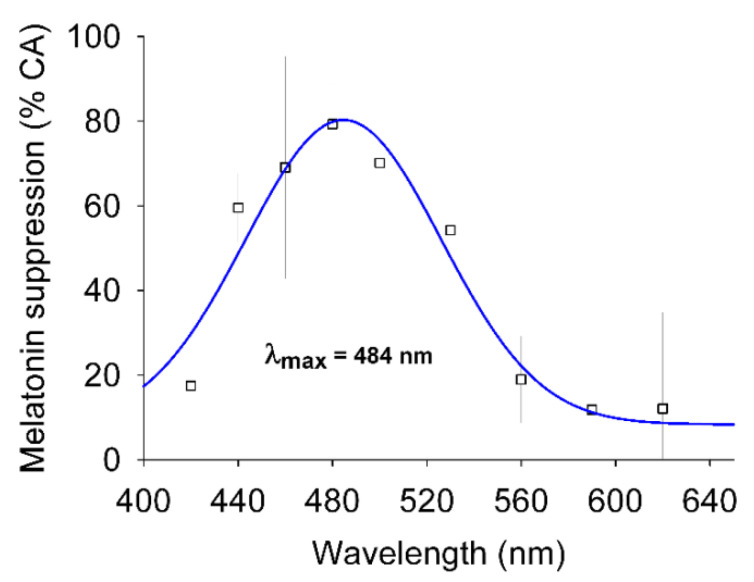
**Action spectrum of acute melatonin suppression by light in humans.** Wavelength-dependent melatonin suppression (expressed relative to control-adjusted (CA) melatonin suppression) after 60 min monochromatic light exposures, at night, at 3.6 × 10^13^ photons/cm^2^/s. The fit (solid blue curve) is a four parameter Gaussian model (*R*^2^ = 0.94). Maximum suppression was found at 484 nm (λ_max_). Figure and legend adapted from [29].

The melatonin secretion pattern, considered as one of the best indirect markers of the circadian clock in the SCN [70], has an action spectrum for its suppression by light that peaks at ~480 nm (Figure 1) [27,28,29], which is the same as that of ipRGCs sensitivity [71,72]. Phase-shifting responses mediated by the master circadian clock are also more sensitive to short wavelengths of 460–470 nm when compared to medium wavelengths of light at 555 nm [30] or long wavelength light at 600 nm [31]. However, although it is likely to peak also at ~480 nm, the spectral sensitivity function for the human phase-shifting response is yet to be determined.

#### 1.2.2. Impact of Duration of Light Exposure

**Figure 2:**
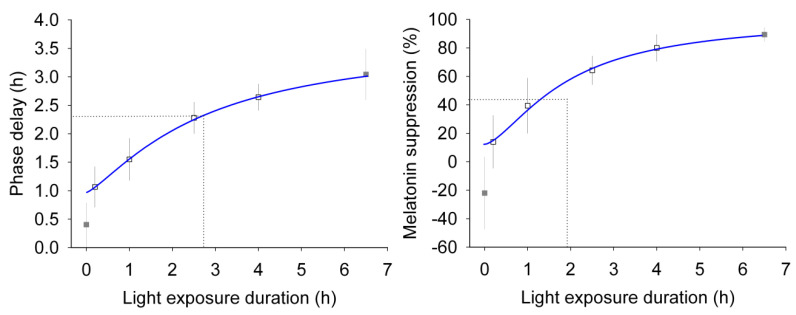
**Duration–response curves of melatonin phase shifts (left) and suppression (right) in humans.** Open symbols represent responses to light exposure durations of 0.2 h, 1 h, 2.5 h, and 4.0 h respectively. Filled symbols represent a dim background control and a 6.5 polychromatic white light exposure [42]. The fits (solid blue curve) are a 4-parameter logistic model. Predicted half-maximum values are shown by the dotted lines. Figures and legend adapted from [32].

Light exposure duration plays also a determinant role. Chang et al. [32] studied the impact of a 10,000 lux light exposure at different durations on circadian phase-shift and melatonin suppression. They found that the responses observed were nonlinear with respect to time (Figure 2). Half of the melatonin phase shift was obtained with 2.7 h duration and half of the maximum value of percentage melatonin suppression with 1.9 h duration. Dewan et al. [33] showed that increasing the duration of the light exposure from 1 to 3 h, but not the intensity (which was between 2000 to 8000 lux), increased the magnitude of light-induced phase delays. Although the light intensities used in the latter study were likely saturating the response, the results overall emphasize that the magnitude of the circadian response depends simultaneously on exposure duration and light intensity. In fact, the relationship between light duration and response is more complex, as sequences of very short light flashes can also induce large phase shifts in humans [43,44] (see also Section 1.2.5).

#### 1.2.3. Effects of Timing of Light Exposure

The response to light is also dependent on the timing of exposure [6,35,36]. Time of day effects have also been shown for acute responses to light such as heart rate and temperature, whereby nighttime exposure significantly increased those responses but not exposures during the daytime period [9]. 

**Figure 3:**
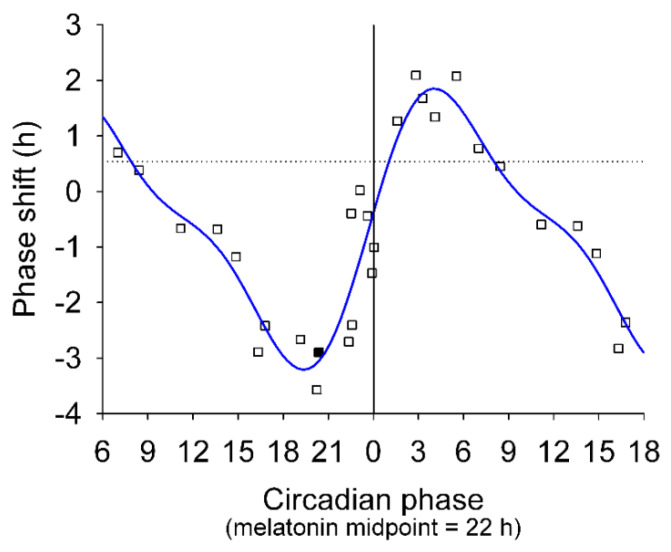
**The human phase response curve to 6.7 h of 10,000 lux polychromatic white light.** Circadian phase advances are shown as positive, and circadian phase delays as negative values on the *y*-axis. They are plotted against the timing of the center of light exposure relative to the plasma melatonin midpoint on the pre-stimulus constant routine (defined to be at circadian phase hour 22), with the core body temperature minimum assumed to occur 2 h later, at circadian phase hour 0 (at 05:30 on average). The solid blue curve is the dual harmonic function fitted through all the individual data points. The horizontal dotted line indicates the anticipated 0.54 h average delay drift of the pacemaker between the pre- and post-stimulus phase assessments. The open squares are data obtained from plasma melatonin samples, the filled square represents salivary melatonin data for one subject. Figure and legend adapted from [6].

The SCN generates an endogenous circadian rhythm, and the magnitude and direction of the effects of light exposure (e.g., on melatonin secretion, core body temperature, cortisol) have been demonstrated to depend on the phase of the circadian cycle [6]. With 6.7 h polychromatic white light exposure at 10,000 lux, a maximum phase delay of 3.6 h and phase advance of 2.01 h was obtained by Khalsa et al. [6] (Figure 3). With 6.5 h of monochromatic light exposure at 480 nm light (2.8 × 10^13^ photons/cm^2^/s), almost the same curve was observed at much lower illuminance levels (11.2 lux), suggesting that shorter-wavelength light of 480 nm is accounting almost entirely for light resetting of the circadian clock [35]. Light exposure administered in the morning (i.e., after the core body temperature minimum, on average after 05:00) phase advances the circadian system, whereas light exposure in the evening/night (on average after 17:00) phase delays the phase of the circadian clock. 

#### 1.2.4. Effects of Light Intensity

**Figure 4:**
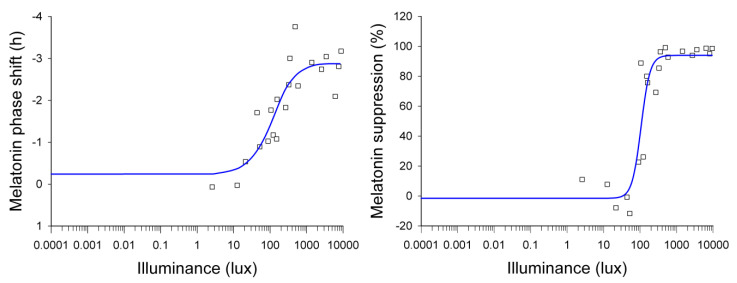
**Illuminance response curves to a 6.5 h polychromatic white light exposure for melatonin phase shift (left) and melatonin suppression (right) in humans.** Individual subjects are indicated by the open squares, the solid blue line shows the sigmoidal curve fitting. Note that the curves and x-axes have been extended to include response levels at very low light intensities. The reader is referred to Figure 5 to compare the melanopic illuminance-response model (1.5 h light exposure) with the illuminance-response model here (6.5 h light exposure). Figures and legend adapted from [38].

**Figure 5:**
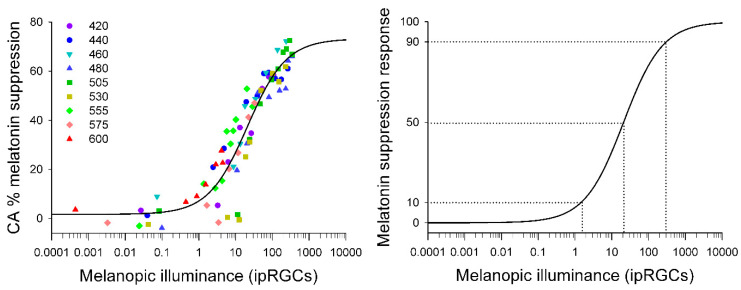
**Melatonin suppression threshold and saturation as a function of melanopic lux, in humans.** **Left**: In colored points, the control-adjusted percentage melatonin suppression for the nine wavelengths used in Brainard et al. (2001, 2008) [27,74]. Light intensities used in those two studies are expressed in melanopic illuminance (melanopic lux). **Right**: Melanopic illuminance-response model of the normalized melatonin suppression response in humans. The horizontal dotted lines correspond to 10% (initiation), 50% (half-maximum response), and 90% (saturation) of the suppression response following 1.5 h exposure to monochromatic lights at night. The initiation threshold for melatonin suppression corresponds to ~1.5 melanopic lux, the half-maximum at ~21 melanopic lux, and saturation begins at 305 melanopic lux. Solid lines represent the sigmoidal fitting. Figure and legend adapted from [40].

Light-dependent circadian responses are also sensitive to the intensity of the light exposure [37,38,40]. Using a 6.5 h (polychromatic white light) exposure centered 3.5 h before minimum core body temperature at a range of light intensities (3–9100 lux), Zeitzer and colleagues generated sigmoidal dose-response curves for melatonin phase-shift and melatonin suppression (Figure 4) [38]. That study found that melatonin phase shift response saturated at ~550 lux (half-maximum at ~120 lux), with no measurable response observed below ~15 lux, consistent with the results by Boivin and colleagues [73]. For melatonin suppression, saturation was found at ~200 lux (half-maximum at ~105 lux), with no measurable response to light below ~30 lux. Melatonin suppression in response to 18 different office lighting conditions (30 min exposure) revealed a dose–response relationship best predicted by the melanopic lux of the lighting [39]. More recent results by Prayag et al. (2019a) [40], show much lower thresholds, with a calculated measurable response at 1.5 melanopic lux (which corresponds to 1.5–9 photopic lux) and saturation at 305 melanopic lux (1.5 h exposure) (Figure 5).

Note on light measurement: in order to measure and compare the biological effects of light, it is recommended to use absolute metrics (irradiance, photon flux), measured on a vertical plane at the eye level, as they are absolute (radiometric) measures and not weighted to the sensitivity of the human photopic luminosity function (as lux does). Another metric (‘melanopic lux’) was recently proposed [75] which allows integration of spectrum and intensity of any light source, accounts for lens transmission (standard observer), and is an estimate of the effective illuminance perceived by ipRGCs (melanopsin). More recently, a new international standard (CIE S 026/E:2018, [76]) recommends to use melanopic irradiance as the best and simplest metric to model human NIF responses in most real-life conditions. For the melatonin suppression response, Prayag et al. [40] showed that melanopic irradiance can be used as a simple metric to model the response and account for most of the suppression, in consonance with the recommendation.

#### 1.2.5. Effects of Temporal Patterns of Light Exposure

Rimmer et al. [41] and Gronfier et al. [42] showed that intermittent bright light pulses can produce a similar response amplitude of the phase shift and suppression of melatonin in humans. Six 15-min pulses of 9500 lux polychromatic bright white light separated by 60 min of dim light produced a similar phase-shift as a continuous 6.5 h exposure at ~9500 lux (with only 23% of the light exposure in duration). Modulated light exposure with bright light pulses (45 min bright light exposures, separated by 60 min of 100 lux) has also been shown to efficiently phase delay (1 h) and entrain the circadian system to longer-than-24 h days [77]. Strikingly, Zeitzer et al. [43] obtained 45 min of phase delay by using sixty 2-ms pulses (corresponding to 120 ms total duration) of 473 lux of white light separated by 60 s of darkness from 02:00 to 03:00. For 2 ms light flashes of 1700–1800 lux an optimal interstimulus interval, which elicited the maximum phase delay response during the night, was determined to be 7.6 s [44]. Revell et al. [78], using 3 episodes of 30-min blue light exposure separated by 15-min intervals obtained average phase shifts close to 1 h. These results confirm that the pattern of light exposure, even with extremely short pulses, has the capacity to strongly influence light-dependent acute and circadian physiology.

#### 1.2.6. Impact of Spatial Distribution of Light Exposure

Acute and circadian effects of different spatial stimulation of the retina have been described in humans. Salivary melatonin suppression by light has been found to be more effective when the nasal retina was exposed compared to the temporal retina [45]. Glickman et al. [46] used a polychromatic white light and observed that exposing the superior area of the retina was less effective in suppressing plasma melatonin compared to the inferior retina. Rüger et al. [47] investigated the influence of the retinal area stimulation in the NIF responses of melatonin suppression, phase delay, subjective sleepiness (KSS), and core body temperature. They found that nasal illumination of the retina resulted in a stronger melatonin suppression compared to temporal illumination. However, KSS scores and core body temperature levels remained similar in all conditions. A circadian phase delay was obtained with nasal illumination but not temporal illumination. Caution must however be exercised as only one or two intensity levels were used in those studies. Results observed might be restricted to those intensities only. Recent characterization of ipRGCs distribution and subtypes in the human retina [20] suggest different ipRGCs functionalities. In rodents, ipRGCs subtypes have been associated with different functions (for review, [21]). Stimulating the retina in an eccentricity-dependent manner, at constant irradiance, resulted in increasing post-illumination pupil response amplitude [48] in humans. Together, these results suggest that the percentage of classical photoreceptors and ipRGCs subtypes stimulated in the human retina can play a role on the magnitude of acute responses to light.

#### 1.2.7. Effects of Prior Light History

Prior light history has been shown to affect the sensitivity of ipRGCs that mediate non-image forming photoreception in mammals [79]. These neurons show light and dark adaptation, i.e., become ‘desensitized’ after exposure to a brief light flash and ‘resensitized’ after time spent in darkness. Human studies indicate that prior light exposure impacts melatonin suppression [49,50,51]. For instance, prior exposure to a week of daytime low light intensity (<200 lux) significantly increased melatonin suppression, as compared to nighttime polychromatic bright light (5000–7000 lux) [49]. Prior light exposure to very dim light also enhanced melatonin suppression when individuals were subsequently exposed to nighttime bright light [50]. Furthermore, dim white light adaptation attenuated melatonin suppression by subsequent exposure to short-wavelength monochromatic light as compared to dark adaptation [51]. In a similar vein, when individuals were exposed to two different light levels (very dim light vs. typical room light) prior to 6.5 h light exposure at night, the prior exposure to very dim light level, as compared to typical room light level, caused a greater phase shift of the melatonin rhythm and acute melatonin suppression [52]. The effects of prior light exposure also impact cognitive brain function, with prior exposure to longer wavelength (~589 nm) as compared to shorter wavelength of light (~461 nm), resulting in increased activation in brain regions associated to executive control [53]. Furthermore, prior illuminance history has also been shown to influence the magnitude of the direct alerting effect of a light stimulus [26]. Importantly, recent findings suggest that prior light exposure can also reduce the subsequent responsivity of the circadian clock, such that bright blue-enriched polychromatic light exposure in the morning, as compared to a control lighting, reduced phase shifts in response to light exposures in the evening [54].

## 2. Impact of Light on Cortical Activity during Wakefulness

**Figure 6:**
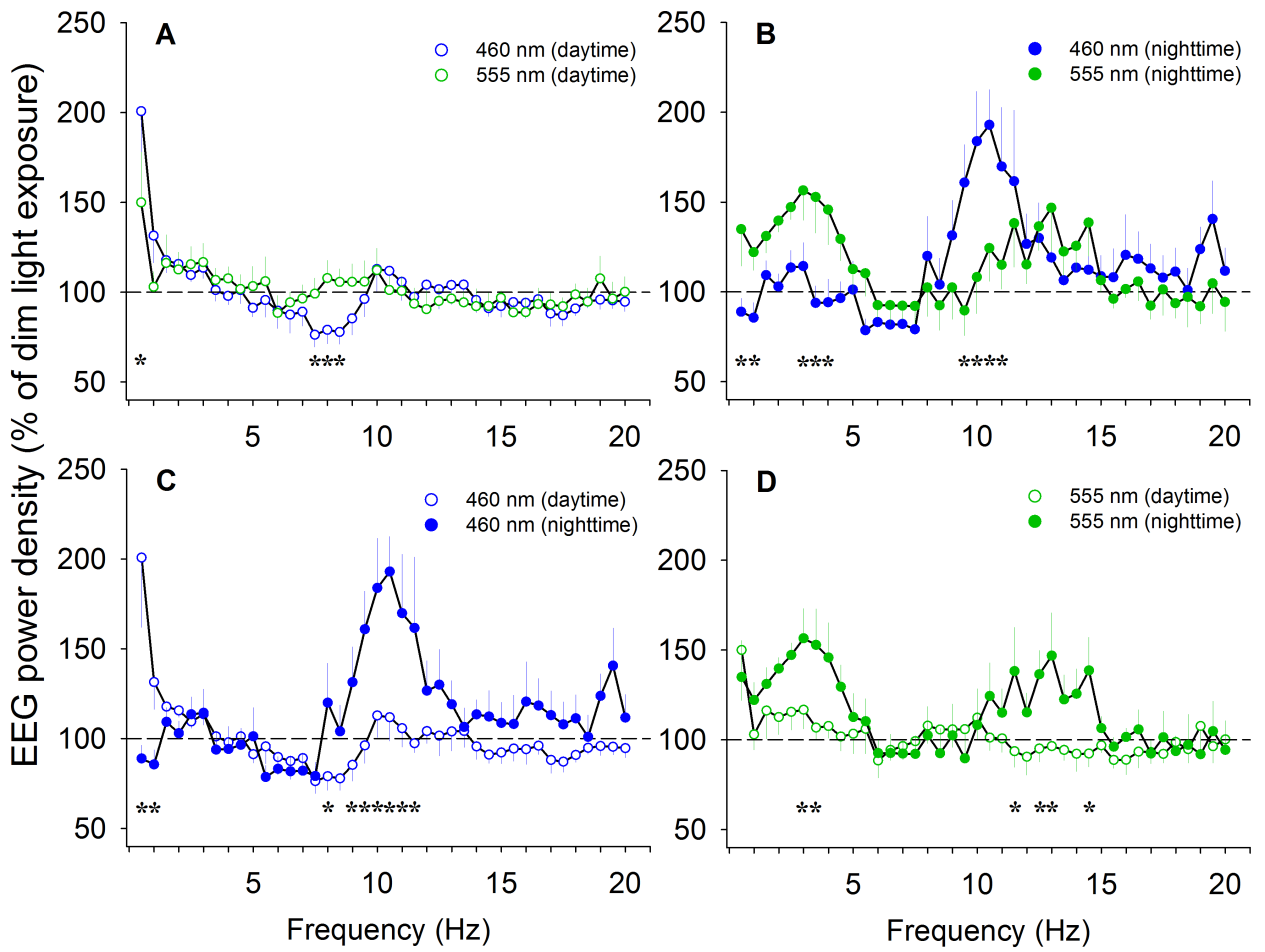
**Variation in the human electroencephalogram (EEG) spectra during daytime and nighttime monochromatic light exposure.** Log-transformed averaged EEG power density during 6.5 h monochromatic light exposure (460 nm and 555 nm, equal photon density of 2.8 × 10^13^ photons/cm^2^/s) was expressed relative to EEG power density during the same clock time 48 h earlier (dim light < 3 lux) under constant routine (100%, dotted line). Average EEG power density in each 0.5 Hz frequency bin was compared between 460 nm and 555 nm exposure during daytime (**A**) and nighttime (**B**). Comparisons between daytime and nighttime for the 460 nm (**C**) and 555 nm (**D**) exposures are also shown. Differences between blue and green monochromatic lights (panels **A**,**B**), and between daytime and nighttime (panels **C**,**D**) are denoted by * (*p* < 0.05). Figure and legend adapted from [11,55].

Bright light is known to impact electrophysiological markers of alertness (faster frequencies of waking EEG) and sleep pressure (delta/theta EEG activity), as well as subjective psychometric measures (assessed via the Psychomotor Vigilance Test; PVT), especially during nighttime exposure. Lockley et al. [55] and Rahman et al. [11] added evidence on this effect of light on the human electroencephalogram in non-sleep deprived conditions (Figure 6). Lockley et al. exposed healthy young subjects to monochromatic light at 460 nm for 6.5 h during nighttime, i.e., starting 1 h before habitual sleep time, and contrasted this exposure with monochromatic light at 555 nm. Rahman et al. carried out an analogous experiment, but during the daytime, starting 4.75 h after habitual waking time. Aggregating results from both studies, it was observed that compared to monochromatic light at 555 nm, monochromatic light at 460 nm reduced EEG delta power (0.5–4 Hz) during the night, reduced EEG theta power (7.5–8.5 Hz) during daytime but not at night, and increased EEG alpha power (9.5–11 Hz) during the night but not during the day. Furthermore, compared to daytime, short-wavelength light increased EEG alpha power (8–11.5 Hz) during nighttime, while green light increased EEG high delta power (3–3.5 Hz) and high alpha power (11.5–14.5 Hz) during the night.

These results highlight the broad-brush effect of the spectral power distribution of light on the human EEG. Interestingly, no differential effect of blue vs. green monochromatic light was observed in EEG delta and theta activity in a blind subject lacking an outer retina contrary to the above results in sighted subjects [18]. In that study, the blind subject had complete visual loss (absence of functional rod and cone responses as assessed by ERG) but ipRGCs were apparently preserved as melatonin suppression and phase entrainment to light were observed. In the wake EEG, an increase in alpha activity (8–12 Hz) occurred with short-wavelength (blue) light, suggesting that only ipRGCs may be involved in the generation of EEG alpha activity response to light.

Under extended wakefulness, light exposure has been shown to influence the EEG either following short exposures of light at high intensities or longer exposures at low intensities. Badia et al. [12] showed that alternating 90-min episodes of bright polychromatic white light with 90-min of darkness increased EEG beta power (15–30 Hz) with each additional light episode. On the other hand, Phipps-Nelson et al. [56] showed that a 6-h exposure to very low intensity monochromatic short-wavelength light prevented the increase of EEG delta (1–4.5 Hz) power. This was observed despite extended wakefulness, which generally results in increased EEG delta power. This same study showed a similar result with EEG theta (4.5–8 Hz) levels, but did not observe enhancement of EEG alpha (8–12.5 Hz) activity, contrary to Lockley and colleagues [55]. Another study reported that a 1-h low-intensity (10–40 lux) monochromatic short-wavelength light exposure after midnight increased EEG beta activity (12–30 Hz) and decreased EEG alpha activity (8–12 Hz) [57]. Similar effects were obtained with 10 lux, but not 40 lux, of monochromatic long-wavelength light in that study. In chronically sleep-deprived subjects, light exposure may not have any impact on the EEG [80]. This study allowed subjects only 8 h of sleep over 48 h, prior to a 3-h light exposure during daytime (either with monochromatic blue or green light) and they found no significant differences between the light conditions on the waking EEG nor on measures of subjective sleepiness, cognitive throughput, or attention-related task.

Collectively, these findings point to a differential impact of light on cortical activity. Faster frequency EEG alpha and beta activities, and markers of alertness were increased, concurrent with a reduction of EEG delta/theta activity, a marker of sleep pressure. High accumulated sleep debt may, however, decrease the responsiveness of the cortex, to light, at least as measured by EEG. Further research is needed to better understand the relationship between sleep pressure, circadian phase, and light.

## 3. Impact of Light on Sleep

The impact of light on human sleep also depends on the characteristics of the light stimulus. Thereby, the same light properties which determine many other NIF responses are also relevant for sleep physiology: i.e., the timing of light exposure (morning/daytime vs. evening/nighttime), duration of exposure(s), light intensity; and finally, the spectral power distribution of the light stimulus, i.e., light sources with shorter vs. longer wavelengths. Most light-induced effects on sleep are indirect, such that light exposure occurred before the sleep episode itself, and can be considered as an effect of light history on sleep. These effects of (prior) light exposure can modulate the circadian responsiveness of NREM/REM sleep across 24-h light/dark exposures, as it was shown in nocturnal rodents [81,82] and in humans [83].

### 3.1. Impact of Timing and Intensity of Light Exposure on Sleep

Previous laboratory studies showed that bright morning light exposure affect sleep timing, duration, and structure [84,85,86]. Bright light exposure very early in the morning (when the preceding sleep episode was curtailed) shortened sleep duration in healthy young subjects during the following night [87]. Other results with bright morning light exposure increased sleep propensity (most likely by advancing circadian phase [88]). Postulating that optimal light exposure is a predictor for consolidated sleep/wake rhythms and high sleep quality implies that light exposure during the day should be sufficiently long and/or bright and/or rich in short wavelengths at 480 nm. In agreement with that proposal, increasing the short-wavelength content of ambient light during daytime was found to increase sleep duration and to correct circadian phase delay in extreme conditions of chronic artificial light exposure (without daylight) [89]. Recent field study data demonstrated the association of higher light intensity exposure during daytime with greater slow-wave sleep (SWS) accumulation on the following night as well as shorter sleep latency to stage 2 sleep [59,60].

Many studies have shown the phase delaying effects of different light exposures in the evening on the following sleep episode [90,91,92]. Brighter light exposures in the evening have become a distinct problem with the progressive use of light emitting devices in the evening, especially in adolescents whose internal sleep–wake preferences are already delayed [93]. Most light emitting devices (LED based) contain a considerable proportion of shorter wavelengths of light, which have potent (negative) effects on sleep initiation and propensity when exposure occurs prior to bedtime [61,62,94,95,96]. It is currently under debate whether artificial light at night (LAN) might pose a health risk to shift worker, e.g., for circadian desynchronization, tumor proliferation, depression, or sleep disorders in humans [97]. Even more, since light at night is penetrating through closed eye lids (while subjects are asleep), high intensity LAN can suppress melatonin and delay circadian phase [68,98], whereas sleep architecture and the number of sleep-wake transitions are not necessarily affected [68]. Nevertheless, constant bed lights at night were associated with less slow-wave sleep (SWS) in young subjects [69] and greater depressive symptoms in a bigger sample of elderly living at home [67].

### 3.2. Impact of Spectral Composition of Light Exposure on Sleep

Exposure to shorter wavelengths of light in the evening/night is known to elicit acute alerting effects and prolonged sleep latencies (as shown in young adults e.g., [61] and young children [99]), reduce REM sleep duration and suppress slow wave activity (SWA) during the first NREM/REM sleep cycle [63,64]. On the other hand, evening exposure to longer wavelengths of light was shown to have sleep promoting effects by shortening sleep onset and increasing sleep duration in rodents [100]. In humans, depleting the short-wavelength content of ambient light for 8 h before bedtime resulted in increased EEG delta-theta activity and reduced melatonin suppression prior to bedtime [58].

More recently, it was also demonstrated in humans that filtering shorter wavelengths of light [65] or using orange/red light exposure [54,66] in the evening increased sleep duration [54,65], sleep propensity [66], without suppressing melatonin [54,65], as shown by several studies in humans including those with new approaches of spectral tuning [58] and metameric light [101]. The exact sleep-promoting mechanisms of longer wavelength light exposure in the evening still need to be elucidated. A different mode of action by melanopsin-dependent neuronal projections on the SCN and the VLPO might play a role, as proposed in mice [100,102]. Taken together, the different properties of light exposure across the 24-h day can lead to changes in sleep timing, sleep propensity, sleep architecture and sleep EEG power spectra. The first goal hereby should be to reduce health risks induced by light and consider specific and/or individual needs, such as in patients with ophthalmic disorders or psychiatric diseases. Future studies and applications should also address comprehensive integration of various qualities of lighting (and darkness) across the 24-h light-dark cycle to support and synergize therapeutic interventions.

## 4. Conclusions

Here, we addressed how light exerts powerful NIF effects on the circadian timing system and the human sleep–wake cycle, primarily through light properties such as intensity, duration, timing, wavelengths, and prior light history. The last decades have seen an exponential surge of our understanding on how targeted light exposure can enhance NIF, circadian, and sleep-wake processes, with direct translational relevance such as, for example, how light during the day improves subsequent sleep quality. Research has also shown that, on the opposite, inadequate light exposure (e.g., too much blue-enriched light) at inappropriate circadian times (e.g., before bedtime) can seriously alter sleep/circadian physiology and lead to disorders in chronic circumstances. In addition, recent work [103] has shown that acute melatonin suppression and circadian responses to light are specific and not proxies of each other. This suggests that NIF responses might not share a single sensitivity function to light and thus follow different illuminance-response and duration–response curves [34]. Ultimately, knowledge on how light affects sleep and wakefulness can improve light settings at home and at the workplace to improve health and well-being and optimize treatments of chronobiological disorders.

## 5. Outlook and Future Directions

Based on a large body of evidence about the importance of sleep for health, future research (in the laboratory and in the field) should systematically address how light can promote health, high waking quality at daytime, and good sleep at night, as well as how improper light exposure may lead to disorders and diseases. Light hygiene should be promoted, and light exposure paradigms and light therapy should be used to prevent/counteract circadian and sleep disruptions in different populations and patient groups. In that context, it will be helpful to identify the effects of daytime exposure on sleep duration and quality. Studies set out to evaluate proper light exposure in children and adolescents to avoid delayed sleep and consequences of LED screen use before bedtime are needed to understand those phenomena. Finally, whether light exposure before bedtime with certain spectral compositions of light could be sleep promoting needs further evaluation.

## Figures and Tables

**Table 1:** Overview of references.

Section	Key References
*Differences in spectral sensitivity to light*	Spectral sensitivity of (i) melatonin suppression by light (Brainard et al. [27]; Thapan et al. [28]; Cajochen et al. [8]; Najjar et al. [29]) (ii) circadian phase-shifting (Lockley et al. [30]; Revell et al. [31])
*Impact of duration of light exposure*	Duration response curves for (i) melatonin suppression and circadian phase-shifting (Chang et al. [32]; Dewan et al. [33]) (ii) EEG, distal to proximal temperature gradient (DPG), heart rate (Prayag et al. [34])
*Effects of timing of light exposure*	Phase response curves (Khalsa et al. [6]; Rüger et al. [35]; St Hilaire et al. [36])
*Effects of light intensity/irradiance*	Illuminance response curves for (i) subjective alertness (KSS), EEG (5–9 Hz), slow-eye movements (Cajochen et al. [37]) (ii) melatonin suppression and circadian phase-shifting (Zeitzer et al. [38]) (iii) melatonin suppression as a function of melanopic illuminance (Nowozin et al. [39]; Prayag et al. [40])
*Effects of patterns of light exposure*	(i) Intermittent bright light and circadian phase-shifting (Rimmer et al. [41]; Gronfier et al. [42]) (ii) Response to milliseconds flashes of light (Zeitzer et al. [43]; Zeitzer & Najjar, [44])
*Impact of spatial distribution of light exposure*	(i) melatonin suppression (Visser et al. [45]; Glickman et al. [46]; Rüger et al. [47]) (ii) pupillary light reflex (Joyce et al. [48])
*Effects of prior light history*	Influence of light history on non-visual responses (Hébert et al. [49]; Smith et al. [50]; Jasser et al. [51]; Chang et al. [52]; Chellappa et al. [53]; Münch et al. [54])
*Impact of light on cortical activity during wakefulness*	Influence of bright light and short-wavelength light on the waking EEG (Badia et al. [12]; Lockley et al. [55]; Phipps-Nelson et al. [56]; Figueiro et al. [57]; Rahman et al. [11,58])
*Impact of light on sleep*	(i) Impact of brighter light during daytime increases SWS accumulation during the following night (Wams et al. [59]; Nowozin et al. [60]) (ii) Effects of short-wavelength light, including from LED devices, before bedtime on sleepiness (e.g., van der Lely et al. [61]), sleep onset (e.g., Chang et al. [62]), REM sleep, SWS and sleep architecture (Münch et al. [63]; Chellappa et al. [64]) (iii) Impact of longer wavelengths light prior to sleep on sleep promotion (Münch et al. [54]; Ostrin et al. [65]; van der Meijden et al [66]) (iv) Impact of light during sleep on melatonin suppression (Obayashi et al. [67]; Zeitzer et al. [68]) and SWS (Cho et al. [69])
